# The Effectiveness of Intermittent Fasting to Reduce Body Mass Index and Glucose Metabolism: A Systematic Review and Meta-Analysis

**DOI:** 10.3390/jcm8101645

**Published:** 2019-10-09

**Authors:** Yongin Cho, Namki Hong, Kyung-won Kim, Sung joon Cho, Minyoung Lee, Yeon-hee Lee, Yong-ho Lee, Eun Seok Kang, Bong-Soo Cha, Byung-Wan Lee

**Affiliations:** 1 Department of Endocrinology and Metabolism, Inha University School of Medicine, Incheon 22332, Korea; 2 Graduate School, Yonsei University College of Medicine, Seoul 03722, Korea; 3 Department of Internal Medicine, Yonsei University College of Medicine, Seoul 03722, Korea

**Keywords:** intermittent fasting, glucose metabolism, insulin resistance, body mass index

## Abstract

The effects of an intermittent fasting diet (IFD) in the general population are still controversial. In this study, we aimed to systematically evaluate the effectiveness of an IFD to reduce body mass index and glucose metabolism in the general population without diabetes mellitus. Cochrane, PubMed, and Embase databases were searched to identify randomized controlled trials and controlled clinical trials that compared an IFD with a regular diet or a continuous calorie restriction diet. The effectiveness of an IFD was estimated by the weighted mean difference (WMD) for several variables associated with glucometabolic parameters including body mass index (BMI) and fasting glucose. The pooled mean differences of outcomes were calculated using a random effects model. From 2814 studies identified through a literature search, we finally selected 12 articles (545 participants). Compared with a control diet, an IFD was associated with a significant decline in BMI (WMD, −0.75 kg/m^2^; 95% CI, −1.44 to −0.06), fasting glucose level (WMD, −4.16 mg/dL; 95% CI, −6.92 to −1.40), and homeostatic model assessment of insulin resistance (WMD, −0.54; 95% CI, −1.05 to −0.03). Fat mass (WMD, −0.98 kg; 95% CI, −2.32 to 0.36) tended to decrease in the IFD group with a significant increase in adiponectin (WMD, 1008.9 ng/mL; 95% CI, 140.5 to 1877.3) and a decrease in leptin (WMD, −0.51 ng/mL; 95% CI, −0.77 to −0.24) levels. An IFD may provide a significant metabolic benefit by improving glycemic control, insulin resistance, and adipokine concentration with a reduction of BMI in adults.

## 1. Introduction

Calorie restriction (CR) is known not only to reduce body weight but also to improve various cardiovascular risk factors [1]. Prolonged CR has been shown to reduce fat mass with significant weight loss. An improvement of insulin resistance was also identified after CR [2]. Mechanisms of CR-mediated improvement of glucose metabolism are not fully elucidated, but possibly involve significant alterations in insulin sensitivity of skeletal muscle [3], along with the reduction of fat mass.

Nevertheless, there are many difficulties in sustaining daily CR [4]. As an alternative diet intervention to CR, weight control through intermittent fasting (IF) has been proposed. A variety of diets have been used to study the effects of IF [5], including alternate-day fasting (ADF, consuming no calories on fasting days) [6], alternate-day modified fasting (ADMF, consuming less than 25% of baseline energy needs on fasting days) [7,8], time-restricted fasting (TRF, restricting food intake to specific time periods of the day) [9], and periodic fasting (PF, fasting only one to two day per week) [10]. In addition to the effect on weight loss, many studies have shown that through IF we can expect a powerful effect on improvement of glucose metabolism by lowering insulin resistance [11], improvement of systemic inflammatory diseases [12,13,14,15], protection against neurodegeneration, and even expansion of the life span [16].

In a recent study, an IF diet (IFD) improved insulin sensitivity even when the supplied calories were the same as those of the control group, without significant change in body weight [17]. This shows that IF, itself, can be beneficial to glucose metabolism independent of body weight changes, however, contrary to other results, in some controlled, randomized crossover trials, IF did not improve glucose and lipid metabolism [18,19]. Likewise, there were also conflicting results in the effect of weight reduction. Although some studies reported significant weight reduction in an IFD group [7,20], several other trials did not report a clinically meaningful weight reduction [18,21].

As seen from the varied and inconsistent findings of previous studies, many hurdles based on the characteristics of the diet intervention study, such as non-standardized dietary control of the patient, low compliance, and the number of participants due to the difficulty of conducting a study covering an entire period, and the difficulty of setting the control group, make it difficult to identify the beneficial effects of an IFD. Furthermore, several previous meta-analysis studies on the glucometabolic effect of an IFD have also shown conflicting results [22,23], which is important for establishing diet recommendations for the general population including the population with obesity or prediabetes. Thus, this study aimed to investigate the effectiveness of IF on weight loss and glucose metabolism by analyzing the effect size of previous studies among the general population without diabetes mellitus (DM).

## 2. Experimental Section

### 2.1. Data Sources and Search Strategies 

We searched articles using Cochrane (from inception to Nov 2018), PubMed (from inception to Nov 2018), and Embase (from inception to Nov 2018) published before November 15, 2018. The terms used in the literature search were “intermittent fasting,” “Ramadan diet”, “Ramadan fasting”, “time-restricted fasting”, “time-restricted feeding”, “alternate fasting”, “periodic fasting”, “periodic diet”, “reduced meal frequency”, “alternate-day fasting” and “alternate-day modified fasting” for intervention terms. These search terms were combined with “OR”. In addition, “normal human”, “adult”, “patient”, “human”, “obesity”, “diabetes mellitus”, “diabetes”, “metabolic syndrome” and “obese” were also combined with “OR” as terms for the objects. The abovementioned two term groups were combined with “AND”. Languages were limited to English. This study was registered before the data analysis (Prospero CRD42019125120). This was a literature-based descriptive study, and therefore approval by the institutional review board or informed consent was not required.

### 2.2. Eligibility Criteria

The inclusion criteria of the literature were as follows: studies on the general population without chronic diseases that could affect glucose metabolism including diabetes mellitus (patients, P); those that studied an IFD (intervention, I); those that compared the control groups, regular diet (RD) or continuous CR diet control without fasting (≥12 h) (comparison, C); and those that described fasting glucose, homeostatic model assessment of insulin resistance (HOMA-IR), total body weight, body mass index (BMI), lean mass, fat mass, leptin, and adiponectin (outcome, O) according to the research question (PICO). Only controlled trials of supervised diet programs were eligible. Studies were limited to interventions which were a minimum 4 weeks in duration. In addition, only studies that presented the mean and standard deviation of the results were selected.

We excluded studies on patients with diabetes mellitus, chronic liver disease, chronic renal disease, and studies on pregnancy (P); those that involved a combination with specific nutrient restriction diet intervention (I); those that compared with a different type of IFD (C); and those with results other than the parameter of glucose metabolism, such as psychological parameter (O). The literature was independently selected by two researchers (Namki Hong and Yongin Cho), and cases of inconsistencies were resolved via a thorough discussion. In consideration of the possibility that some articles could be missed by the above search terms, we further searched the review papers of similar topics to see if there were any papers corresponding to the same PICO. 2.3. Data Extraction

Fasting glucose, HOMA-IR, BMI, fat mass, lean mass, leptin, adiponectin, and total body weight were used as variables for the effect of an IFD and were calculated using the mean, standard deviation, number of participants, as well as baseline and final values. The contents of the literature were examined in detail, and the types of IFD and control diet were coded and presented. If there was a numerical result due to the different control group in the contents of one document, the data were extracted according to categories for the control group and errors resulting from the duplication of the same documents were supplemented using statistical techniques.

### 2.3. Statistical Analysis

To supplement the systematic review, a meta-analysis was performed. STATA 15 (StataCorp LLC, College Station, TX, USA) was used to synthesize the effect sizes of the 12 selected documents. The weighted mean difference (WMD) between the baseline and final measurements of fasting glucose, HOMA-IR, BMI, fat mass, lean mass, leptin, adiponectin, and total body weight according to the intervention were considered as the effect size, and the inverse variance method was used because the values were continuous variables. The random effects model was chosen because of the diversity and heterogeneity of the intervention. Heterogeneity was estimated using the I^2^ statistic across the studies [24]. I^2^ values were interpreted as follows: 0% to 40%, no important heterogeneity; 30% to 60%, moderate; 50% to 90%, substantial; and 75% to 100%, considerable heterogeneity. The heterogeneity of pooled effects between subgroups was calculated using the Cochran Q statistic. A funnel plot method was used to test potential publication bias.

## 3. Results

### 3.1. Search Results and Study Characteristics

**Figure 1:**
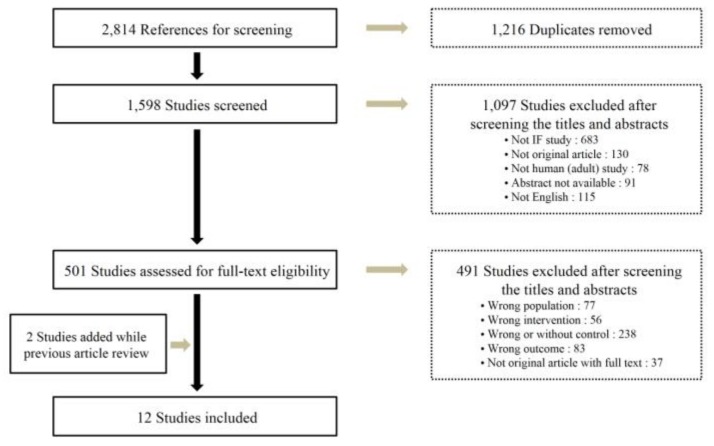
Flowchart of the study selection.

A total of 2814 studies were selected according to the search method of our study. After eliminating 1216 duplicate documents, the titles and abstracts of 1598 studies were reviewed, and 1097 papers that had no relevance with the original review according to the PICO were excluded. Among 501 studies selected by reviewing the titles and abstracts, 12 studies were finally included in this study (Figure 1). The total number of participants was 545 (261 in the intervention group and 284 in the control group; 210 or 38.5% men and 335 or 61.5% women). The general characteristics of the included studies are summarized in Table 1.

### 3.2. Effectiveness of Intermittent Fasting on BMI and Body Weight 

**Figure 2:**
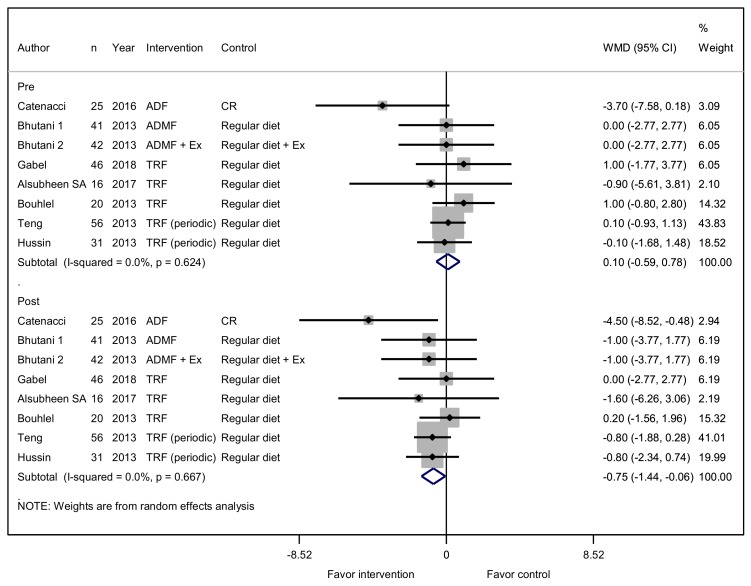
Effect of intermittent fasting (IF) versus a non-fasting control on body mass index (changes from baseline) in adults without chronic metabolic disease. The squares indicate the study-specific outcome estimates, and the size of the squares corresponds to the study’s weight in the meta-analysis. Horizontal lines denote the range of the 95% confidence interval. The diamonds indicate pooled estimates. Weights are from random effects analysis.

A total of eight studies reported on the effects of an IFD on BMI. Seven studies assessed diet intervention only, and one of the studies assessed diet intervention with exercise, which was equally assessed in the control group. No difference was observed between the two groups for BMI at baseline (WMD, 0.10 kg/m^2^; 95% CI, −0.59 to 0.78; *p* = 0.783). After the diet intervention, BMI was significantly lower in the IFD group by 0.75 kg/m^2^ (95% CI, −1.44 to −0.06; *p* = 0.033; Figure 2). The IFD was also associated with a trend towards reducing body weight. A total of 10 studies reported on the effects of an IFD on body weight. All details of the changes in body weight were presented in kilograms (kg). No difference in body weight at baseline was observed between the two groups (WMD, 0.24 kg; 95% CI, −3.18 to 3.66; *p* = 0.889). The weight of the participants was lower in the IFD group by 1.94 kg (95% CI, −5.20 to 1.31; *p* = 0.241), without statistical significance (Supplementary Figure S1).

### 3.3. Effectiveness of Intermittent Fasting on Glycemic Control

**Figure 3:**
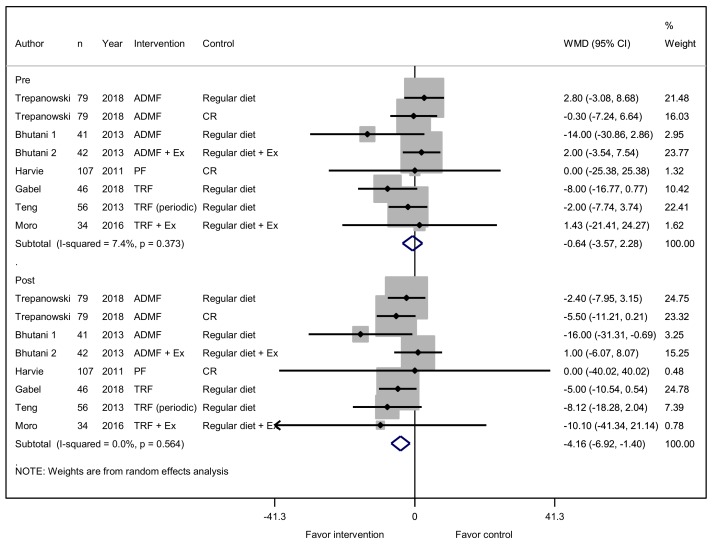
Effect of IF versus non-fasting control on fasting blood glucose (changes from baseline) in adults without chronic metabolic disease.

A total of eight studies reported on the effectiveness of an IFD to reduce fasting glucose levels. Six studies assessed an IFD only, and two other studies assessed an IFD with exercise. No difference was observed between the two groups in fasting glucose at baseline (WMD, −0.64 mg/dL; 95% CI, −3.57 to 2.28; *p* = 0.666). After intervention, a significant decrease in fasting glucose level (WMD, −4.16 mg/dL; 95% CI, −6.92 to −1.40; *p* = 0.003) was confirmed (Figure 3).

**Figure 4:**
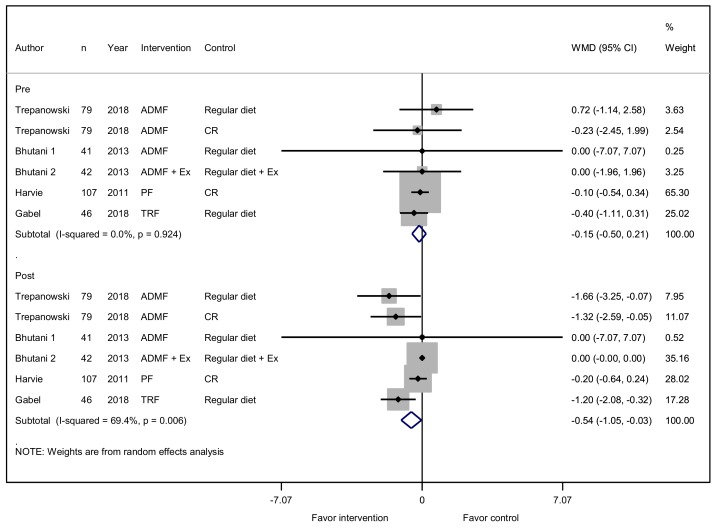
Effect of IF versus non-fasting control on homeostatic model assessment of insulin resistance (HOMA-IR) (changes from baseline) in adults without chronic metabolic disease

An IFD was also associated with lowered HOMA-IR. A total of six studies reported on the effectiveness of an IFD to reduce HOMA-IR levels. The HOMA-IR levels of the participants were lower in the IFD group by 0.54 (95% CI, −1.05 to −0.03; *p* = 0.038) after diet intervention (Figure 4).

### 3.4. Effect of Intermittent Fasting on Lean and Fat Mass and Circulating Leptin and Adiponectin

Lean mass did not show significant differences between the two groups, both in the baseline (WMD, −1.11 kg; 95% CI, −2.62 to 0.41; *p* = 0.153) and after diet intervention (WMD, −1.26 kg; 95% CI, −2.78 to 0.26; *p* = 0.105; Supplementary Figure S2). Although without statistical significance, an IFD was associated with a trend towards reducing fat mass. The fat mass was higher in the IFD group at baseline (WMD, 0.37 kg; 95% CI, −0.99 to 1.72; *p* = 0.596), however, it was lower after intervention (WMD, −0.98; 95% CI, −2.32 to 0.36; *p* = 0.151; Supplementary Figure S3).

Although there was no significant change in fat mass after an IFD, an IFD was associated with an increase in adiponectin level (WMD, 1008.87 ng/mL; 95% CI, 140.45 to 1877.29; *p* = 0.023; Supplementary Figure S4) and a decrease in leptin level (WMD, −0.51 ng/mL; 95% CI, −0.77 to −0.24; *p* < 0.001; Supplementary Figure S5) without the difference in the baseline.

## 4. Discussion

The gold standard management of obesity or overweight is 20% to 30% CR along with a comprehensive lifestyle modification [31], however, rebound weight gain is a real problem of weight reduction management in clinical situations. Recently, an IFD has been gaining popularity as an alternative strategy for achieving and maintaining weight reduction. Considering the discrepancy of IFD effectiveness on glucose metabolism and the lack of evidence in meta-analyses, we conducted a systematic search and selected 12 studies. On the basis of this meta-analysis, we confirmed an improvement of fasting blood glucose and insulin resistance through IFD as compared with a non-fasting control group. This seems to be related to a decrease in the BMI and a leptin level reduction, as well as an increase in adiponectin level. Lean mass was relatively conserved in the IFD group, however, no significant weight reduction was identified. In previous studies, the overall weight reduction of both an IFD and a daily CR (by 15% to 60% of the usual caloric intake every day) were similar between two groups [32]. In this study, the analysis of total body weight included an additional paper with higher degree of continuous CR diet as a control group, which explains why there was a statistically different discrepancy between total body weight and BMI in this study.

With respect to IFD effectiveness on glucose metabolism, many challenges remain. In some previous studies, the effect of IFD on blood glucose was not significant [33], and there were different findings according to the characteristics of participants. A 3% to 6% decrease in fasting glucose was observed in patients with prediabetes [30,34], whereas no significant effect on fasting glucose concentration was observed in healthy individuals [7]. In this study, we included all studies of participants with or without prediabetes, because the definition of prediabetes was often unclear in previous studies. As a result, we found an improvement in blood glucose (fasting glucose level; WMD, −4.16 mg/dL; 95% CI, −6.92 to −1.40; *p* = 0.003) and insulin resistance (HOMA-IR; WMD, −0.54; 95% CI, −1.05 to −0.03; *p* = 0.038) in the general population with the inclusion of some obese with prediabetes population groups. Insulin resistance is associated with DM development [35]. Therefore, IFD might be more beneficial to patients with high insulin resistance who are likely to progress to DM.

The beneficial effects of an IFD including effects on glucose metabolism are often regarded to be driven by reductions in body weight and/or body fat [5]. Previous IFD studies that adopted ADMF or ADF diet intervention with a relatively high degree of CR resulted in clinically meaningful reductions in body weight [6,7], whereas a relatively small (<5.0 kg) weight reduction was achieved in IFD studies adopting a TRF diet with small degree of CR [21,25]. In addition, no significant weight loss was observed in studies that adjusted the fasting time while maintaining the total calorie intake [17]. Thus, the main pathophysiologic mechanism of weight loss through an IFD is likely to be a reduction in the total calories consumed. In this study, no significant weight loss was observed in the IFD group as compared to the non-fasting control group. The main explanation for this result might be the mixed characteristics of the control group including both continuous CR and RD groups. Nevertheless, a tendency toward weight reduction was observed in the IFD group (WMD, −1.94 kg; 95% CI −5.20 to 1.31; *p* = 0.241), and a significant decrease was observed in the analysis with BMI (WMD, −0.75 kg/m^2^; 95% CI, −1.44 to −0.06; *p* = 0.033).

Approximately one-fourth to one-third of weight reduction is known to be of lean tissue during continuous CR, however, lean mass has been preserved in most previous studies under an IF diet intervention [25]. Similarly, in this study, lean mass did not show a significant difference before (WMD, −1.11 kg; 95% CI, −2.62 to 0.41; *p* = 0.153) and after (WMD, −1.26 kg; 95% CI, −2.78 to 0.26; *p* = 0.105) the diet intervention. Balancing maximizing loss of body fat and minimizing loss of lean mass is one of the important pathophysiologic approaches to maintaining physical function and reducing insulin resistance. The conservation of lean mass may have contributed to the maximization of HOMA-IR reduction.

Although fat mass reduction during an IFD was not statistically significant (at baseline WMD, 0.37 kg; 95% CI, −0.99 to 1.72; *p* = 0.596, after intervention WMD, −0.98; 95% CI, −2.32 to 0.36; *p* = 0.151), a significant increase in adiponectin level (WMD, 1008.87 ng/mL; 95% CI, 140.45 to 1877.29; *p* = 0.023) and a decrease in leptin level (WMD, −0.51 ng/mL; 95% CI, −0.77 to −0.24; *p* < 0.001) were observed. Leptin and adiponectin are adipose tissue derived hormones and are associated with glucose metabolism. Lower leptin concentrations are generally accompanied by a reduction of fat mass [36]. Adiponectin levels are negatively correlated with insulin resistance, fat mass, and central fat distribution [37], and based on this analysis, we suggest that an IFD seems to be associated with an improved distribution of central fat.

Considering the merits of an IFD and its mechanism of different results as compared to continuous CR, attention has been given to the importance of the fasting time duration in previous studies. After 12 hours of fasting, lipolysis starts in fat tissue and then shifts metabolism from fat storage and lipid synthesis to mobilization of fat as a form of fatty acid-derived ketones [5]. This can be expected to reduce fat mass and improve insulin resistance. With similar calorie intake reductions during continuous CR, this difference in fasting time affects the mass and distribution of fat tissue, finally making a difference in glucose metabolism, as well as adipokine level changes. In addition, as an expected advantage of IFD in comparison to continuous CR, an IFD can be sustained with higher compliance and could have a more significant effect on fat mass reduction and insulin resistance throughout the fasting period.

The association of circadian rhythm with an IFD is an important issue. In a recent study, increased risk of cardiovascular disease was reported in a population without or with only a simple breakfast [38]. Skipping breakfast is associated with an increase in the release of stress hormones [39]. In addition, there was a study showing that significantly improved weight loss and improved insulin resistance were observed in a group that consumed more calories in the morning and less in the evening, in conditions with the similar calorie restriction [40]. The type of interventions in the studies included in this analysis were quite diverse, and there was a limitation to further analysis. Randomized controlled trials under refined conditions could be helpful to clarify the association with circadian rhythm.

This study had some other limitations. First, only studies that presented the mean and standard deviation of the baseline and final results with a control group were selected. For this reason, the number of participants included in the analysis was small. Second, we only included the general population without chronic metabolic disease, and therefore we could not determine through this analysis whether an IFD is effective in patients with chronic metabolic disease such as diabetes mellitus. The intervention group included various types of IF, and the degree of calorie restriction varied accordingly. The non-fasting control groups also ranged from RD to continuous CR. Despite this diversity, the subgroup analysis was limited due to the insufficient number of studies. With respect to the geometric features of participants, including ethnicity, several studies showed heterogeneous characteristics and others did not provided sufficient information, and therefore we could not conduct further analysis. Third, since the selected studies all had interventions of short-term (four to 24 weeks) duration, it is limited to expect long-term effects of IFD.

In some respects, these limitations may serve as strengths. This study was conducted to analyze only refined studies with a control group. The participants of this study represent the general population with a wide range of BMI, and therefore confirm the IF effectiveness on general population without chronic metabolic disease. Through this study, we found that IF improves glucose metabolism through lipolysis by confirming the difference in BMI, HOMA-IR, leptin level, and adiponectin concentration among the participants. Through the results of this analysis, we can expect an improvement of fat distribution during an IFD, however, further studies are required to clarify this benefit of IFD.

In conclusion, IF significantly improves glycemic control and insulin resistance with a reduction in BMI, a decrease in leptin level, and an increase in adiponectin concentration in the general population without chronic metabolic disease.

## Figures and Tables

**Table 1:** Characteristics of the studies included in the meta-analysis.

Author	Year	Design	Duration (wk)	Intervention	Intervention Detail	Fasting Time of Intervention Group (h)	Calorie Reduction (From Baseline, %)	Control (Without Fasting)	Control Detail	Calorie Reduction (From Baseline, %)
Trepanowski et al. 1 [8]	2018	66 women, 13 men (aged 18–65 y, overweight to obese, BMI 25–40 kg/m^2^)	24	ADMF	alternating every 24 h between consuming 25% or 125% of energy needs	24	21.0	RD	consuming 100% of needs every day	Not shown
Trepanowski et al. 2 [8]	2018	66 women, 13 men (aged 18–65 y, overweight to obese, BMI 25–40 kg/m^2^)	24	ADMF	alternating every 24 h between consuming 25% or 125% of energy needs	24	21.0	CR	Consuming 75% of needs every day	24.0
Catenacci et al. [6]	2016	19 women, 6 men (aged 18–55 y, obese, BMI over 30 kg/m^2^)	8	ADF	fast on alternate days, fasting day, at libitum	24	47.0	CR	400 kcal/d deficit from estimated energy requirements	28.0
Gabel et al. [9]	2018	41 women, 5 men (aged 25–65 y, obese, BMI 30–45 kg/m^2^)	12	TRF	ad libitum feeding between 10 am to 6pm, fasting between 6pm to 10am	16	20.3	RD	not to change their eating or physical activity habits	Not shown
Moro et al. [25]	2016	34 men (aged 29.21 ± 3.8 y, weighted 84.6 ± 6.2 kg)	8	TRF + Ex	1 p.m., 4 p.m., 8 p.m. feeding	16	3.2	RD + Ex	8 a.m., 1 p.m., 8 p.m. feeding	Not shown
Bhutani et al. 1 [20]	2013	80 women, 3 men (aged 25–65 y, obese, BMI 30–39.9 kg/m^2^)	12	ADMF	25% of their baseline energy needs on the fast day (24 h), ad libitum on feed day, 12 p.m. to 2 p.m. meals on fast day, 3 days/wk	22	Not shown (450 kcal/d reduction)	RD	maintain regular food habits	Not shown
Bhutani et al. 2 [20]	2013	80 women, 3 men (aged 25–65 y, obese, BMI 30–39.9 kg/m^2^)	12	ADMF + Ex	25% of their baseline energy needs on the fast day (24 h), ad libitum on feed day, 12 p.m. to 2 p.m. meals on fast day, 3 days/wk	22	Not shown (450 kcal/d reduction)	RD + Ex	maintain regular food habits + Ex	Not shown
Teng et al. [26]	2013	56 men (aged 50–70 y, BMI 23–29.9 kg/m^2^)	12	TRF (periodic)	reduction of 300–500 kcal/d from participants baseline energy intake combined with two days of Muslim Sunnah fasting per weeks	13, approximately	19.2	RD	maintain regular food habits	Not shown
Alsubheen et al. [27]	2017	16 men	4	TRF	Muslim Ramadan	13	16.0	RD	maintain regular food habits	7.8
Bouhlel et al. [28]	2013	20 men (aged 20 y)	4	TRF	Muslim Ramadan	Muslim Sunnah fasting	10.9	RD	maintain regular food habits	Not shown
Hussin et al. [29]	2013	31 men (aged 50–70 y, BMI 23–29.9 kg/m^2^)	12	TRF (periodic)	reduction of 300–500 kcal/d from participants baseline energy intake combined with two days of Muslim Sunnah fasting per week	Muslim Sunnah fasting	10.3	RD	maintain regular food habits	Not shown
Harvie et al. [10]	2011	107 women (aged 30–45 y)	24	PF	VLCD for 2 days per week	unknown	29.7	CR	25% restriction	20.4
Tinsley et al. [21]	2017	18 men	8	TRF (periodic) + Ex	On non-workout days (four days per week), consume all calories in any four hour window between 4 p.m. and midnight / unrestricted on RT day	20	13.8	RD + Ex	RT	20.3
Varady et al. [30]	2013	22 women, 8 men (aged 35–63 y, BMI 20–29.9 kg/m^2^)	12	ADMF	25% of their baseline energy needs on the fast day (24 h), ad libitum on feed day, 12 p.m. to 2 p.m. meals on fast day	22	38.0	RD	Ad libitum	Not shown

## Supplementary Materials

The following are available online at https://www.mdpi.com/2077-0383/8/10/1645/s1, Figure S1: Effect of IF versus non-fasting control on body weight (changes from baseline) in adults without chronic metabolic disease, Figure S2: Effect of IF versus non-fasting control on lean mass (changes from baseline) in adults without chronic metabolic disease, Figure S3: Effect of IF versus non-fasting control on fat mass (changes from baseline) in adults without chronic metabolic disease, Figure S4: Effect of IF versus non-fasting control on adiponectin level (changes from baseline) in adults without chronic metabolic disease; and Figure S5: Effect of IF versus non-fasting control on leptin concentration (changes from baseline) in adults without chronic metabolic disease.
