# Supplementary material for: The Effectiveness of Intermittent Fasting to Reduce Body Mass Index and Glucose Metabolism: A Systematic Review and Meta-Analysis
Source: J Clin Med. 2019 Oct 9;8(10):1645. doi: 10.3390/jcm8101645 (PMC6832593; doi:10.3390/jcm8101645)
Supplement: Supplementary file 1 [file jcm-08-01645-s001.pdf]

Supplementary Material

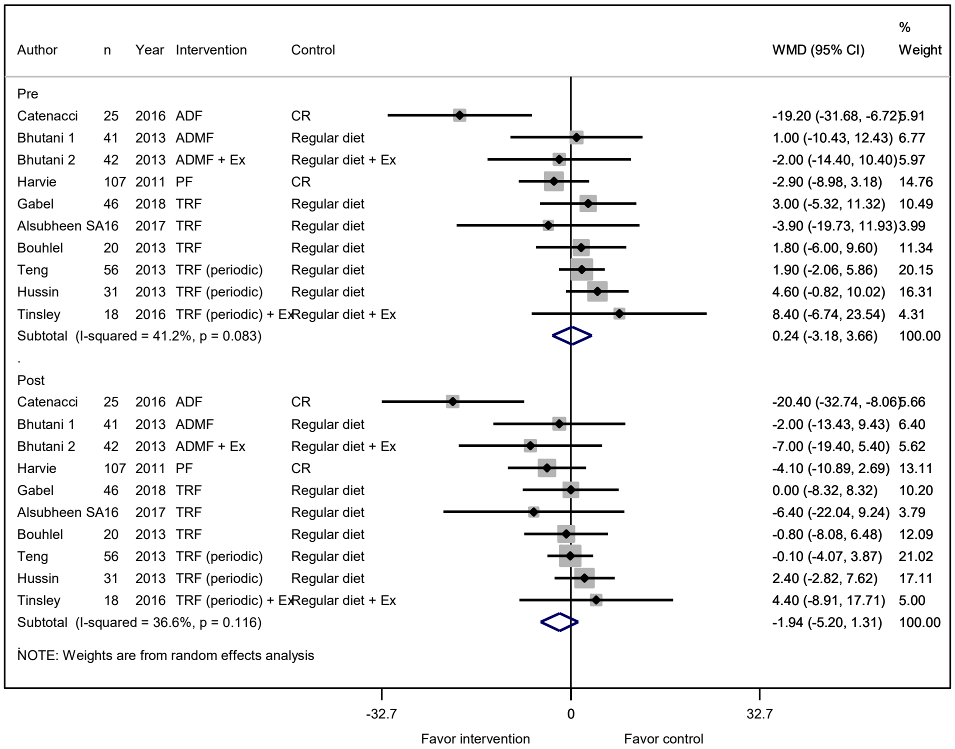

Supplementary Figure 1. Effect of intermittent fasting (IF) versus non-fasting control on body weight (changes from baseline) in adults without chronic metabolic disease. The squares indicate the study-specific outcome estimates, and the size of the squares corresponds to the study's weight in the meta-analysis. Horizontal lines denote the range of the 95% confidence interval. The diamonds indicate pooled estimates. Weights are from random effects analysis.

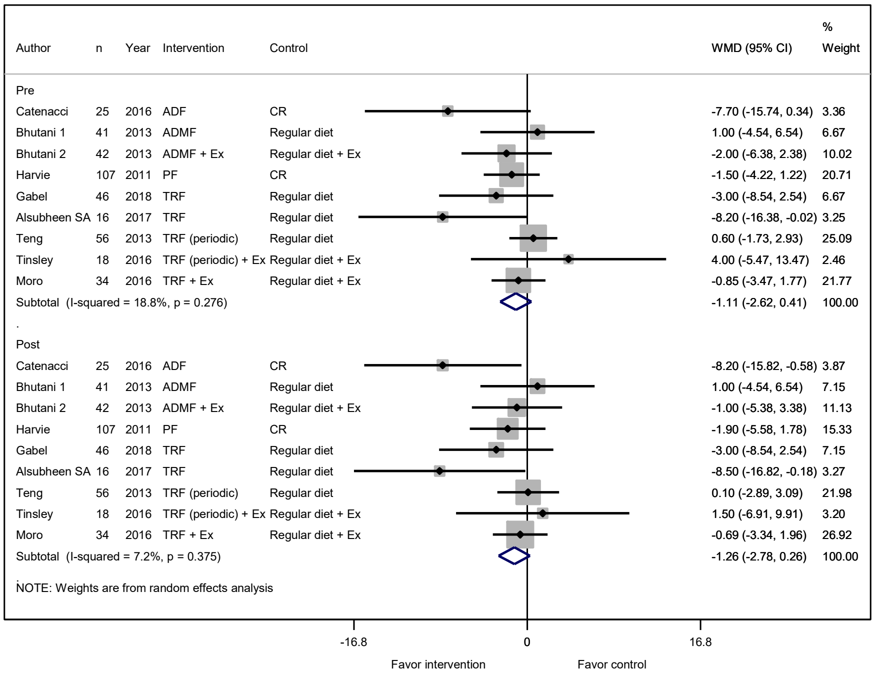

Supplementary Figure 2. Effect of IF versus non-fasting control on lean mass (changes from baseline) in adults without chronic metabolic disease.

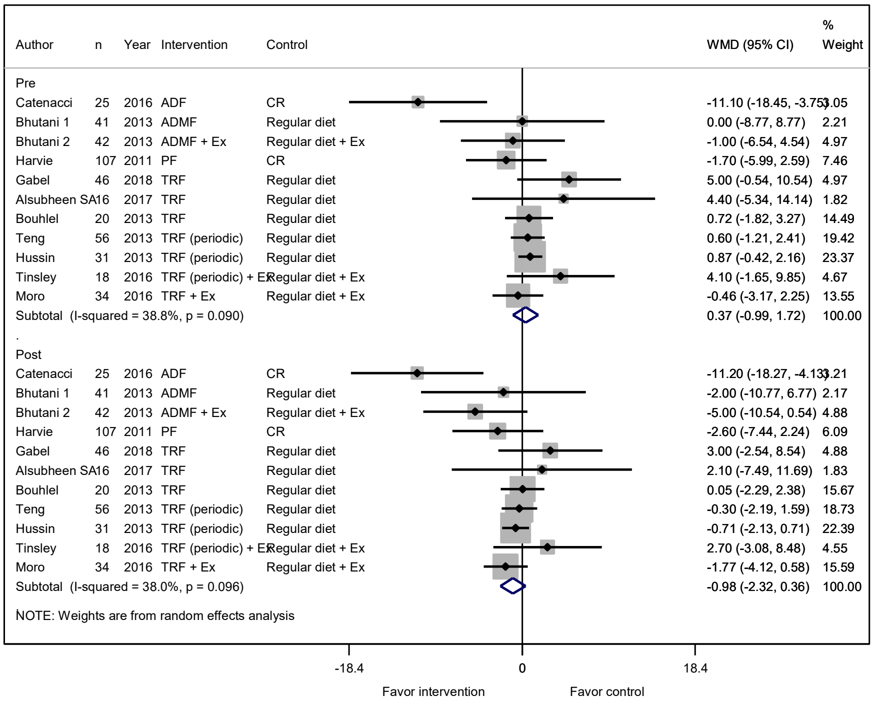

Supplementary Figure 3. Effect of IF versus non-fasting control on fat mass (changes from baseline) in adults without chronic metabolic disease.

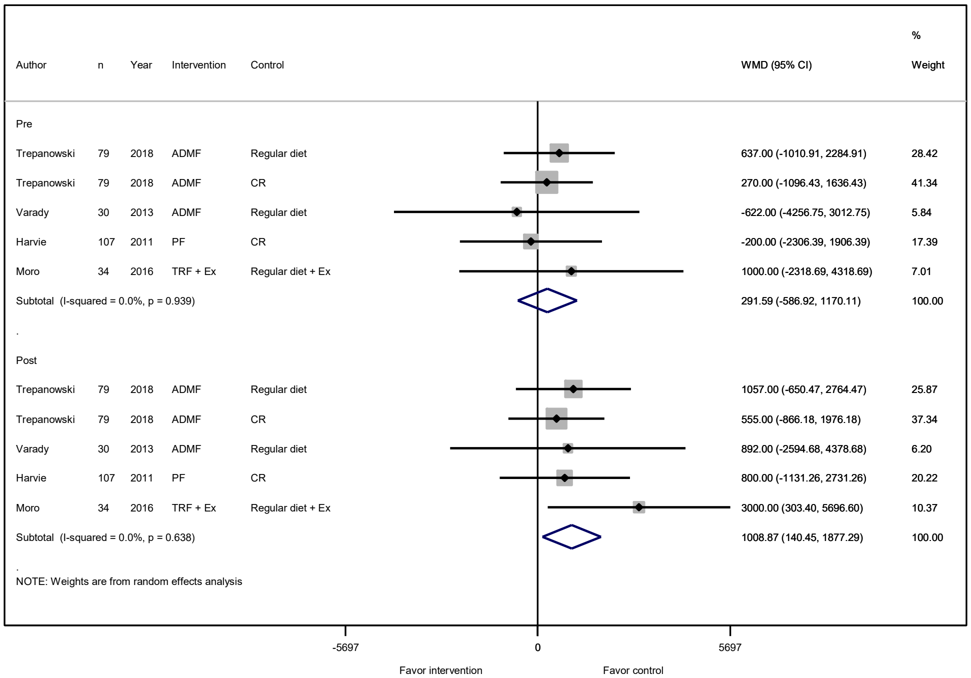

Supplementary Figure 4. Effect of IF versus non-fasting control on adiponectin concentration (changes from baseline) in adults without chronic metabolic disease.

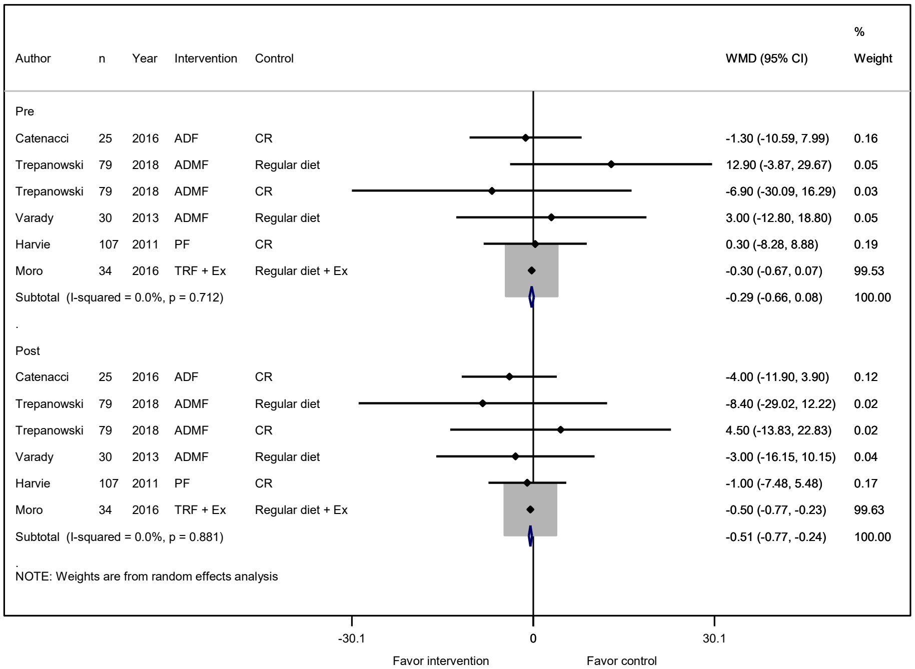

Supplementary Figure 5. Effect of IF versus non-fasting control on leptin level (changes from baseline) in adults without chronic metabolic disease.
